# The outcome of intravenous and inhalation anesthesia after pancreatic cancer resection: a retrospective study

**DOI:** 10.1186/s12871-022-01703-8

**Published:** 2022-05-30

**Authors:** Jie Ren, Junli Wang, Jingwen Chen, Yue Ma, Yutong Yang, Ming Wei, Yu Wang, Liping Wang

**Affiliations:** Department of Anesthesiology, Harbin Medical University Cancer Hospital, No.150 Haping Rd., Nangang District, Harbin, 150081 China

**Keywords:** TIVA, INHA, SIPTW, Pancreatic cancer, Overall survival, Disease-free survival

## Abstract

**Background:**

Different types of anesthesia may affect cancer patient’s outcomes, we compared the overall survival (OS) and disease-free survival (DFS) of patients with pancreatic cancer under total intravenous and inhalation anesthesia.

**Methods:**

The authors collected the electronic medical records of patients who had accepted at a pancreatectomy from January 1, 2010 to December 31, 2016. Patients respectively received total intravenous anesthesia (TIVA) or inhalational anesthesia (INHA). Stabilized inverse probability of treatment weighting (SIPTW)was used to minimize differences. Kaplan–Meier survival was established to analyze the influence of sort of anesthesia on disease-free and overall survival. We compare the effects of each sort of anesthesia on cancer recurrence or metastasis and all-cause mortality.

**Results:**

A total of 463 patients who had undergone pancreatic cancer resection were collected in this study, of which 421 patients were available (TIVA group, *n* = 114 INHA group, *n* = 307). After SIPTW there were no significant differences between the two groups in disease-free survival (hazard ratio, 1.01, 95%CI, 0.78 to 1.29, *P* = 0.959) or overall survival (hazard ratio, 1.11, 95%CI, 0.87 to 1.42, *P* = 0.405).

**Conclusions:**

In conclusion, the present study showed no significant difference in overall survival and disease-free survival between total intravenous anesthesia and volatile anesthesia.

## Synopsis

Intravenous anesthesia can prevent postoperative tumour metastases via several methods. In this study, we aimed to assess the interaction of intravenous anaesthesia and long-term outcome after pancreatic cancer.

## Background

Pancreatic cancer (PC) is one of the most common gastrointestinal malignancies with a five-year survival rate of only 10% [1]. Surgery is a common treatment for pancreatic cancer, which is diagnosed late due to a lack of effective screening methods [2]. Although the tumor is removed by surgery, it also inevitably enters the blood circulation or lymphatic circulation and migrates to distant organs, causing tumor recurrence and metastasis [3]. In addition, surgical trauma and the use of anesthetic drugs in the perioperative period can inhibit the body's anti-tumor immunity [4]. The effect of different anesthetic drugs on tumor cells and cancer patients has always been a research hotspot [5, 6].

Studies have shown that propofol can exert anti-tumor effects through various mechanisms, including inhibiting tumor viability, inhibiting tumor progression, inhibiting cancer cell invasion, etc [7, 8]. In contrast, sevoflurane exhibited immunosuppression and tumorigenesis through a number of mechanisms, including suppression of natural killer (NK) cell activity and lymphocyte function, which induce proliferation, apoptosis, and invasion of cancer cells [9, 10].

In the prior research, we have proved an association between total intravenous anesthesia (TIVA) and the improvement of overall survival for gastric cancer (GC)patients who underwent resection [11]. Therefore, we continue the relevant retrospective study to compare the overall survival and disease-free survival of patients after pancreatic cancer surgery with propofol-based TIVA and sevoflurane-based INHA.

## Methods

### Patient identification and exclusion

Cases of PC were identified from the records of patients and patients admitted to the hospital for cases resection between January 1, 2010 to December 31, 2016. Patients with metastasis, emergency operations, laparoscopic procedures and incomplete clinical data were excluded [11, 12]. Patients who experienced anesthesia and postoperative pathologies with PC were included. Medical records for all of the included patients were obtained, and the data were extracted by researchers who were not involved in the study or data analysis.

### Anesthesia technique and grouping method

In both groups, anesthesia was induced with midazolam 0.05–0.15 mg/kg, 0.5 μg/kg fentanyl, and 1–2.5 mg/kg propofol [11]. Patients were divided into TIVA and INHA groups according to different anesthesia techniques. In the TIVA group, anesthesia was maintained with propofol and remifentanil. In the INHA group, anesthesia was maintained with sevoflurane and remifentanil. The postoperative pain management methods were the same in the two groups and neither has undergone epidural anesthesia.

### Indicator and data

The statuses of patients up to November 30, 2019 were determined from medical records and causes of death were record. We obtained the following information: demographic data, cancer stage, degree of differentiation, American Society of Anesthesiologists (ASA) grade, duration of surgery, primary diagnosis, transfusion, preoperative or postoperative adjuvant chemotherapy, and/or radiation therapy were received [6, 13]. Cancer stage was assessed based on the 8th edition of American Joint Committee on Cancer (AJCC) Cancer Staging Manual [12].The degrees of differentiation included well differentiated and poor differentiated. The primary diagnosis included pancreatic head cancer, pancreatic body or tail cancer. Types of surgery included distal pancreatectomy, pancreaticoduodenectomy and other kinds of pancreatectomy. Survival time was measured from the date of pancreatectomy to death or to the last followed-up before November 30, 2019.

The primary endpoint of this study was overall survival (OS), which was defined as the period from the patient’s date of surgery to the time of death. The secondary endpoint was disease-free survival (DFS), which was defined as the interval between the date of surgery and the date of tumor recurrence and metastasis or death.

### Statistical approach

The cases with unqualified data were excluded from the final analysis, we analyzed the cases that meet the requirements in this study. Fisher’s exact text or χ2 test was used to evaluate the associations between categorical variables. T-tests or Manne Whitney U tests were used to compare continuous variables between patient groups. Categorical data was expressed as n (%) and analysed with the χ2 test, continuous data was expressed as the mean (standard deviation, SD) or median [interquartile range], and two independent samples were analysed with the T-test [6, 13].

The Kaplan Meier method was used to calculate OS and DFS. Cox proportional hazards regression models were used to compare risk factors between the different groups by using univariate models. Significant variables in univariate analysis and clinically significant variables were entered into multivariate analysis. Propensity score matching was used to reduce the difference between groups, which would inevitably reduce the sample size. Thus, we chose stabilized inverse probability of treatment weighting (SIPTW) to make a good balance [14]. These variables were entered in our propensity model: age, sex, ASA physical status, duration of surgery, degree of differentiation, cancer stage, surgery type, hypertension, smoke, blood transfusion, diabetes, drink, tumor location and adjuvant treatment. All analyses were performed using R software version 4.1.2(R Foundation for Statistical Computing, Austria). We used the package “survival” for the Cox regression analysis and package “IPW survival” for the stabilized inverse probability of treatment weighting. Forest plot was built by “forestplot” package and *p*-value < 0.05 was considered statistically significant.

## Results

**Fig. 1:**
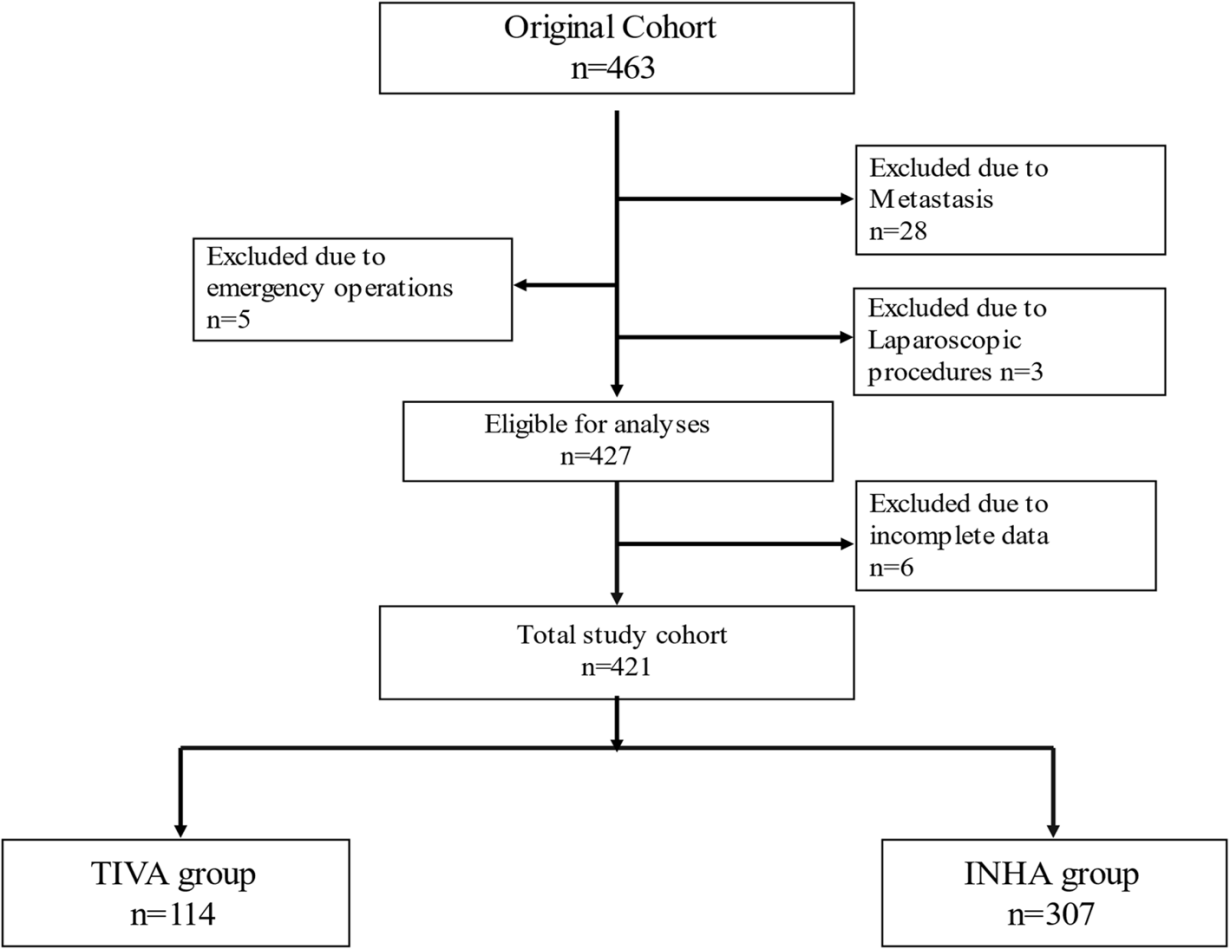
Patient identification and exclusion. INHA, Inhalational anesthesia; TIVA, Total intravenous anesthesia

This retrospective analysis of 463 patients who underwent pancreatectomy for PC were enrolled in this study. After the inclusion criteria were applied, 114 patients were in the propofol intravenous group and 307 patients were in the inhalation group (Fig. 1). The SIPTW procedure was performed to adjust for imbalances in these retrospective settings. After stabilized inverse probability of treatment weighting, the sum of weights was 113.6 in the TIVA group, and307.2 in the INHA group. All standardized mean differences (SMD)for the study variables were less than 0.1 (Table 1).

**Table 1:** Patient Characteristics for before SIPTW adjustment and after SIPTW adjustment

Varible	Before SIPTW adjustment			After SIPTW adjustment ^a^			
	INHA	TIVA	*P*	INHA	TIVA	*P*	SMD ^b^
				Sum of weight	Sum of weight		
	*n* = 307	*n* = 114		= 307.2	= 113.6		
Sex (%)			0.667			0.995	0.001
Female	131 (42.7)	52 (45.6)		133.9 (43.6)	49.6 (43.6)		
Male	176 (57.3)	62 (54.4)		173.2 (56.4)	64.0 (56.4)		
Age	57.00	57.00	0.430	57.00	57.00	0.772	0.018
(median-IQR, year)	[51.0,63.0]	[49.25, 62.00]		[51.0,62.0]	[50.00, 62.00]		
BMI	23.44	22.86	0.305	23.41	23.04	0.898	0.066
(median-IQR, kg/m2)	[21.34,25.81]	[21.43,25.16]		[21.26,25.65]	[21.63,25.94]		
Smoke(%)			0.538			0.995	0.001
No	171 (55.7)	68 (59.6)		175.1 (57.0)	64.8 (57.0)		
Yes	136 (44.3)	46 (40.4)		132.1 (43.0)	48.8 (43.0)		
Drink (%)			0.858			0.824	0.024
No	236 (76.9)	86 (75.4)		235.9 (76.8)	88.4 (77.8)		
Yes	71 (23.1)	28 (24.6)		71.3 (23.2)	25.2 (22.2)		
Hypertension (%)			1.000			0.940	0.008
No	252 (82.1)	93 (81.6)		251.5 (81.9)	92.7 (81.6)		
Yes	55 (17.9)	21 (18.4)		55.6 (18.1)	20.9 (18.4)		
Diabetes (%)			0.760			0.858	0.019
No	264 (86.0)	96 (84.2)		263.0 (85.6)	98.0 (86.3)		
Yes	43 (14.0)	18 (15.8)		44.1 (14.4)	15.6 (13.7)		
Adjuvant treatment (%)			0.832			0.901	0.014
No	213 (69.4)	81 (71.1)		215.0 (70.0)	80.2 (70.6)		
Yes	94 (30.6)	33 (28.9)		92.2 (30.0)	33.4 (29.4)		
Blood transfusion (%)			0.355			0.861	0.020
No	185 (60.3)	75 (65.8)		189.4 (61.7)	68.9 (60.7)		
Yes	122 (39.7)	39 (34.2)		117.7 (38.3)	44.6 (39.3)		
Duration	4.00	4.00	0.956	4.00	4.00	0.520	0.019
(median-IQR, h)	[3.5, 4.5]	[3.5, 4.5]		[3.5, 4.5]	[3.5, 4.5]		
ASA (%)			0.290			0.996	0.009
I	7 (2.3)	4 (3.5)		7.9 (2.6)	2.8 (2.5)		
II	294 (95.8)	105 (92.1)		291.0 (94.8)	107.7 (94.8)		
III	6(2.0)	5 (4.4)		8.2(2.7)	3.1(2.7)		
Tumour location (%)			0.425			0.963	0.005
Head	224 (73.0)	78 (68.4)		220.0(71.6)	81.1(71.4)		
Tail	83 (27.0)	36 (31.6)		87.2(28.4)	32.5(28.6)		
TMN (%)			0.372			0.986	0.019
I	158 (51.5)	51 (44.7)		152.1 (49.5)	56.2 (49.5)		
II	124 (40.4)	50 (43.9)		126.9 (41.3)	46.4 (40.8)		
III	25 (8.1)	13 (11.4)		28.2 (9.2)	11.0 (9.7)		
Surgery type (%)			0.660			0.999	0.005
Distal pancreatectomy	89 (29.0)	38 (33.3)		93.0 (30.3)	34.6 (30.5)		
Pancreaticoduodenectomy	2 (0.7)	1 (0.9)		2.2 (0.7)	0.8 (0.7)		
Other	216 (70.4)	75 (65.8)		212.0 (69.0)	78.1 (68.8)		
Degree of differentiation(%)			1.000			0.806	0.027
Poor	115 (37.5)	43 (37.7)		114.3 (37.2)	40.8 (35.9)		
Well	192 (62.5)	71 (62.3)		192.9 (62.8)	72.8 (64.1)		

*Abbreviations*: *IQR* Inter-quartile range; Cancer stages: stage I: T1, N0, M0/T2, N0, M0/T1, N1, M0; stage II: T3, N0, M0/T4a, N1, M0/T3, N1, M0/T2, N2, M0/T1, N3, M0; stage III: T2, N3, M0/T3, N2, M0/T3, N3, M0/T4a, N2, M0/T4a, N3, M0/any T4b, any N, M0; stage IV: any T, any N, M1.*ASA* American Society of Anesthesiologists, *BMI* Body mass index, *INHA* Inhalational anesthesia, *TIVA* Total intravenous anesthesia, *SIPTW* Stabilized inverse probability of treatment weighting, *SMD* Standardized Mean Difference

*Note:*.^a^ Because the weighted values were presented, the number of patients were not an integer

^b^ Confounding factors were adjusted, SMD < 0.1, *P* > 0.05

**Fig. 2:**
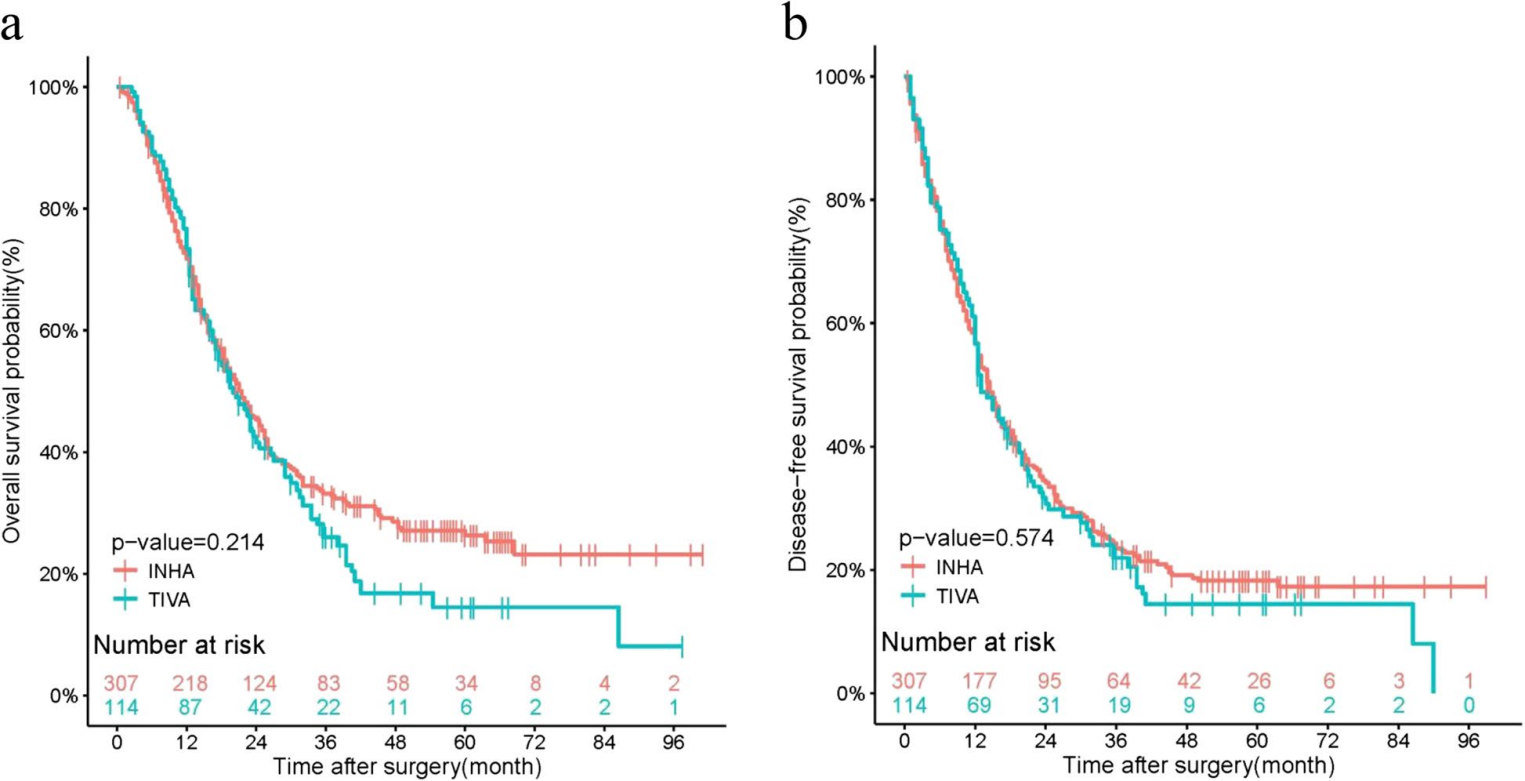
Kaplan–Meier survival curve for overall survival and disease-free survival after SIPTW; SIPTW, stabilized inverse probability of treatment weighting

In this study, the median follow-up time for all patients was 18.5 months (interquartile range, 10.5 to 35.5). TIVA group was 17.5 months (interquartile range,12 to 31.38) and INHA group was 18.5 months (interquartile range,10.0 to 38.25). The Kaplan–Meier survival curves demonstrated the OS rates for 1-year and 3-year in TIVA were 73.4% (95%CI, 65.6% to 82%),26.0%(95%CI,18.4% to 36.7%) and in INHA 71.7%(95%CI, 66.8% to 77.1%), 33.2%(95%CI, 28.0% to 39.2%). The DFS rates for 1-year and 3-year in TIVA were 56.6% (48.0% to 66.8%), 21.9% (95%CI,15.0% to32.0%) and in INHA were 56.7% (95% CI,51.4% to 62.6%), 23.4% (95%CI,19.0% to 28.9%). There was no significant difference in overall survival (*p*-value = 0.214) or disease-free survival (*p*-value = 0.574) between the TIVA group and the INHA group in the SIPTW cohort (Fig. 2a, b).

In the SIPTW cohort, the Cox proportional hazards model for overall survival and disease-free survival were built to evaluate the association between type of anesthesia and overall survival or disease-free survival. Univariate Cox regression revealed no significant association between TIVA and poorer OS (hazard ratio = 1.18,95%CI,0.93 to 1.50, *p* = 0.170) or DFS (hazard ratio = 1.07,95%CI,0.85 to 1.36, *p* = 0.561) when compared with the INHA group (Table 2).

**Table 2:** Univariate analysis of OS and DFS

Varibles	OS(SIPTW)		DFS(SIPTW)	
	HR.CI95	*P*	HR.CI95	*P*
Age	1.02(1.01–1.03)	0.001	1.01(1.00–1.02)	0.020
Sex				
Female	Reference			
Male	0.94(0.75–1.19)	0.612	0.88(0.71–1.09)	0.228
BMI	0.96(0.92–0.99)	0.036	0.98(0.95–1.02)	0.273
Group				
INHA	Reference		Reference	
TIVA	1.18(0.93–1.50)	0.170	1.07(0.85–1.36)	0.561
Smoke				
No	Reference		Reference	
Yes	1.12(0.89–1.42)	0.341	0.90(0.73–1.13)	0.376
Drink				
No	Reference		Reference	
Yes	1.02(0.77–1.34)	0.902	0.90(0.70–1.18)	0.451
Hypertension				
No	Reference		Reference	
Yes	1.12(0.83–1.51)	0.461	0.93(0.71–1.22)	0.584
Diabetes				
No	Reference		Reference	
Yes	0.83(0.57–1.20)	0.317	0.81(0.58–1.13)	0.215
Adjuvant treatment				
No	Reference		Reference	
Yes	0.65(0.50–0.85)	0.001	0.65(0.51–0.84)	< 0.001
Blood transfusion				
No	Reference		Reference	
Yes	1.22(0.97–1.54)	0.091	1.32(1.06–1.65)	0.015
Duration	1.17(1.07–1.28)	0.001	1.12 (1.03–1.23)	0.008
ASA				
I	Reference		Reference	
II	1.78(0.83–3.81)	0.141	2.19(1.04–4.60)	0.039
III	4.67(1.74–12.51)	0.002	7.23(3.08–16.93)	< 0.001
Tumour location				
Head	Reference		Reference	
Tail	0.78(0.59–1.03)	0.077	0.98(0.756–1.26)	0.856
Cancer stage				
I	Reference		Reference	
II	0.99(0.78–1.27)	0.965	1.21(0.97–1.52)	0.098
III	1.18(0.75–1.84)	0.475	1.59(0.99–2.55)	0.057
Surgery type				
Distal pancreatectomy	Reference		Reference	
Other	1.13(0.19–6.60)	0.896	0.73(0.15–3.58)	0.700
Pancreaticoduodenectomy	1.34(0.74–1.76)	0.033	1.11(0.86–1.43)	0.440
Degree of differentiation				
Poor	Reference		Reference	
Well	0.71(0.56–0.91)	0.006	0.71(0.57–0.89)	0.003

*Abbreviations***:***OS* Overall survival, *DFS* Disease-free survival, *SIPTW* Stabilized inverse probability of treatment weighting, *HR* Hazard ratio *CI* Confidence interval, *ASA* American Society of Anesthesiologists

**Fig. 3:**
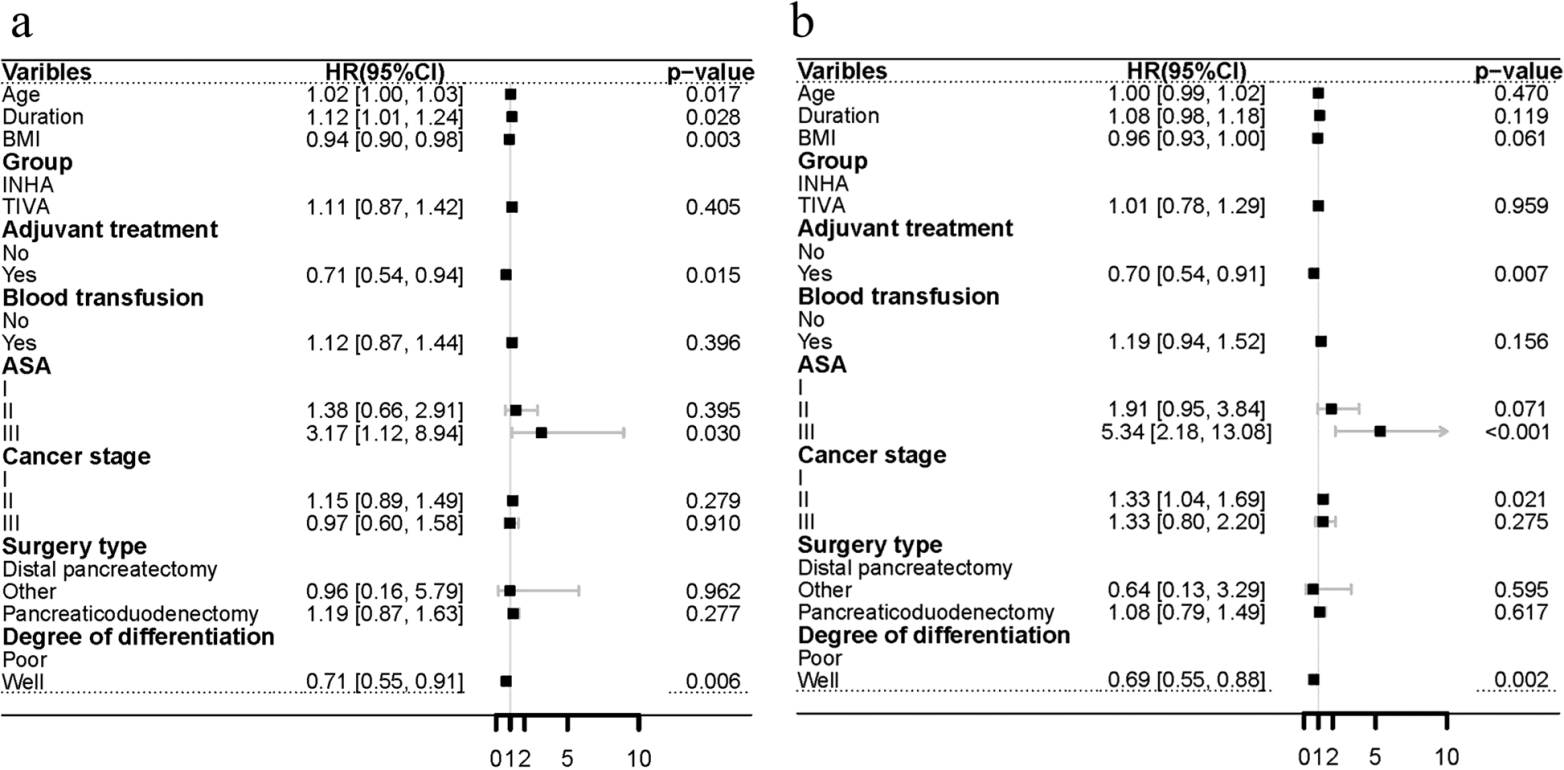
**a** Forest plot for multivariable cox proportional of overall survival after SIPTW **b** Forest plot for multivariable cox proportional of disease-free survival after SIPTW.ASA, American Society of Anesthesiologists; SIPTW, stabilized inverse probability of treatment weighting

In the multivariate Cox model considered that include factors which are *p* < 0.05 in the univariate Cox regression or clinically significant factors. The results were shown in the forest plots that there were no significant between TIVA and IHNA to improve OS (HR = 1.11,95%CI,0.87 to 1.42, *p* = 0.405) or DFS (HR = 1.01,95%CI,0.78 to 1.29, *p* = 0.959) in PC patients. Adjuvant treatment (HR = 0.71,95%CI,0.54 to 0.94, *p* = 0.015, HR = 0.70,95%CI,0.54 to 0.91, *p* = 0.007) and Degree of differentiation (HR = 0.71,95%CI,0.55 to0.91, *p* = 0.006, HR = 0.69,95%CI,0.55 to 0.88 *p* = 0.002) remained statistically significant in improving OS and DFS (Fig. 3a, b).

## Discussion

This study demonstrated no significant correlation between total intravenous anesthesia (TIVA) and better overall survival (OS) in patients who underwent PC surgery. We also found that total intravenous anesthesia did not improve the disease-free survival (DFS) of patients. The results of this study inconsistent with a study that propofol-based TIVA can enhance the prognosis of patients with PC [15].

In recent years, the prognosis of anesthetics for various kinds oftumor patients was investigated. One study found that total intravenous anesthesia based on propofol had a better survival in colon cancer [16]. Another study conducted a retrospective cohort study on gastric cancer surgeries in 2856 patients, and reported improved overall survival in the TIVA group, compared to the inhalation group [11]. In contrast, some retrospective studies proved no difference between the TIVA group and inhalation group for overall survival in digestive cancer surgery [17, 18]. Soltanizadeh et al. [19] also conducted a systematic review pointing out although tumor patients tend to choose TIVA, however, current evidence is not convincing and randomized clinical trials are warranted in order to explore the impact of INHA/TIVA on OS and recurrence-free survival (RFS) after cancer surgery.The available data suggested that the impact of propofol based TIVA on long term outcome of cancer surgery is still controversial currently.

The immunomodulatory effect of anesthetics was considered to be the vital mechanism by which anesthesia affects the prognosis of cancer. Both in vivo and in vitro experiments had shown that inhalation anesthesia inhibits the toxicity of NK cells, which was critical to prevent tumor growth [20, 21]. Furthermore, several studies had shown that volatile anesthetic agents induce upregulation of tumorigenic growth factors, including hypoxia-inducible factor-1 and serum inflammatory factor [22, 23]. Corresponding to propofol, it had been found to enhance the activity of NK cells, reduce tumour inflammatory factors, and have protective and anticancer effects [24, 25]. However, there were also some studies showing that the two anesthetics had little effect on immune cells and the inhibition and activation of the two types of anesthesia drugs may depend on clinical conditions [22, 26].

In our research, we have included more patients than previous study [15], and adopted a new method to reduce the difference between the two groups. Retrospective studies [11, 15] usually used propensity score matching to reduce confounding between groups. Propensity score matching can cause the loss of a large amount of data. IPTW can increase the amount of data several times and increases the false positive rate [14]. SIPTW not only balanced the confounding factors of the two groups of people, but also kept the amount of data indistinguishable from the original [14]. DFS might replace OS as a surrogate endpoint for cancer patients [27], but it had not been proven in pancreatic cancer [28]. OS is still the main criterion for judging the survival of PC patients. Therefore, our study took OS as the main target and DFS as a comparison and supplement.

In the multivariate COX risk proportional regression model, OS was related to age. Adjuvant treatment and degree of differentiation were significantly correlated with OS, which was consistent with the results of previous studies [13, 29]. Obesity led to lower survival rates in pancreatic who underwent pancreatoduodenectomy [30]. Our study confirmed that BMI is associated with OS in pancreatic cancer patients. Age and duration of surgery were also associated with OS. However, age, duration of surgery and BMI had nothing to do with the length of DFS. ASA was often used to assess patient status, it has been proved that patient status was related to metastasis and recurrence [31]. In our study, ASAIII had a worse DFS than ASAI. But the amount of data was too small and the persuasive ability of the data was too weak. In addition, Cancer stage II has worse DFS than Cancer stage I and there was also no correlation between the type of surgery and OS or DFS.

The effect of perioperative blood transfusion on tumor prognosis has always been controversial. In a retrospective study, perioperative blood transfusion was associated with OS and DFS in patients with stage I to III gastric cancer who underwent tumor resection, and the amount of transfusion OS and DFS showed a non-linear dose relationship [32]. Another multicenter regression study found that perioperative blood transfusion among patients undergoing radical rectal cancer surgery was associated with worse OS, not with worse DFS [33]. When restricted to pancreatic cancer, previous studies have found no relationship between blood transfusion and OS [15], another review suggests that there is currently no conclusive evidence that PC is associated with perioperative blood transfusion [34]. Our study verified that OS in pancreatic cancer was not correlated with blood transfusion, and DFS was also not correlated with blood transfusion.

Opioids are an important part of general anesthesia, especially in tumor patients. Although the impact of opioids on the prognosis of tumors was still controversial, opioid-sparing onco-anaesthesia is the way forward [35]. We did not collect perioperative opioid use because literature showed that it did not affect the survival rate of PC [29].There were some inevitable limitations to our study. Firstly, cardiovascular-related diseases were not included which could have influenced the choice of anesthesia, resulting in a statistical bias.Secondly, the sample size was single, which required multi-center and larger clinical data. Thirdly, because of the retrospective study design, it was not possible to measure levels of inflammatory biomarkers that could explain the causal relationship between type of anesthesia used and recurrence of cancer.

In conclusion, the present study revealed no significant difference in overall survival and little difference, if any, in disease-free survival between TIVA and INHA.

## Data Availability

All data generated or analyzed during this study are included in this published article.

## Abbreviations

PC: Pancreatic cancer
NK: Natural killer
GC: Gastric cancer
SIPTW: Stabilized inverse probability of treatment weighting
TIVA: Total intravenous anesthesia
INHA: Inhalational anesthesia
ASA: American Society of Anesthesiologists
AJCC: American Joint Committee on Cancer
DFS: Disease-free survival
OS: Overall survival
